# Adolescents with type 1 diabetes’ perspectives on digital health interventions to enhance health literacy: a qualitative study

**DOI:** 10.3389/fpubh.2024.1340196

**Published:** 2024-02-13

**Authors:** Aurélia Naoko Naef, Nadine Fischbock, Hürrem Tezcan-Güntekin, Volker Eric Amelung

**Affiliations:** ^1^Institute for Epidemiology, Social Medicine and Health Systems Research, Hannover Medical School, Hannover, Germany; ^2^Department of Health and Education, Alice Salomon Hochschule Berlin, Berlin, Germany; ^3^Berlin School of Public Health, Charité Universitätsmedizin Berlin, Berlin, Germany

**Keywords:** digital health, digital interventions, health literacy, adolescents, type 1 diabetes, patient-centered, patient-participation

## Abstract

**Introduction:**

Digital health intervention offers the potential to enhance health literacy, which is crucial for effective diabetes management, especially among adolescents. Diabetes is a major global public health issue, leading to devastating complications and increasing mortality rates. The incidence of type 1 diabetes mellitus (T1DM) is also on the rise, particularly among adolescents, necessitating multisectoral strategies to combat this disease. This study explores the perceptions of adolescents with T1DM in Germany regarding digital health interventions, with the aim of improving healthcare by addressing specific needs and guiding future research.

**Methodology:**

This study employed a qualitative approach using semi-structured individual interviews with adolescents with T1DM (*n* = 20) aged 14 to 18 years old in Germany to explore their perspectives on digital interventions for health literacy promotion. The study adopted content analysis according to Kuckartz et al. and the research followed the Consolidated Criteria for Reporting Qualitative Research (COREQ) checklist. Ethical considerations were paramount and data were rigorously analyzed using coding and iterative processes to ensure data quality and reliability.

**Results:**

The findings indicate that within three prominent domains, namely the utilization of digital health intervention for accessing and comprehending information, facilitating peer-to-peer interactions, and enhancing physician-patient communication and interaction, digital health interventions are either underutilized or insufficiently deployed. In addition, a notable observation is the apparent lack of patient-centered approaches for adolescents with T1DM in relation to digital health interventions and health literacy.

**Conclusion:**

In order to enhance the utilization of digital health interventions and enhance health literacy it is essential to focus on capacity building through a patient-centered approach, to promote digital health literacy, and foster the cultivation of a participatory culture. The outcomes of this study offer valuable insights that can inform practical applications, further research endeavors, and influence policymaking.

## 1 Introduction

Adolescence is a vulnerable period of life characterized by significant physiological and psychosocial changes and a need to gain autonomy from parents (1). When it coincides with the presence of a chronic disease such as type 1 diabetes, the transition into adulthood is made even more challenging (2, 3). Today’s generations of adolescents are known for their massive use of digital tools (4, 5); thus offering an opportunity to integrate digital interventions into the healthcare system to improve the management of their conditions. Diabetes is considered one of the most significant global public health challenges, having evolved into a major worldwide public health concern (6). It not only represents a leading cause of blindness but also contributes to kidney failure, heart attacks, strokes, and lower limb amputations (7). Between 2000 and 2019, diabetes-related mortality rates increased by 3% per age group (7). The incidence rates of Type 1 Diabetes (T1DM) are also on the rise, contributing to the overall increase in diabetes prevalence (8). In Germany, the incidence of T1DM among children and adolescents has shown a consistent increase over the past 30 years (9). Projections predict a rise in case numbers in the coming decades, underscoring the urgency of developing and implementing multisectoral strategies to combat this disease (8). Effective management of this chronic condition demands a comprehensive understanding of both medical and behavioral aspects, highlighting the crucial importance of health literacy within this population, not only for reducing mortality (10), but also for mitigating health inequalities (11). Young individuals afflicted with this condition must be capable of navigating a complex landscape of medical information and making informed healthcare decisions, all while traversing the vulnerable period of adolescence.

Digital health interventions (DHI), such as telehealth, mobile health, messaging systems, mobile applications, gamified support, social platforms, and patient portals (12) have rapidly expanded in recent decades, offering improved disease management (13–17). They hold the potential to enhance health literacy and the management of T1DM among adolescents (18). Digital health and the use of digital technologies have become a global health priority for improving healthcare delivery and chronic disease management. The recent report from the World Health Organization Regional Office for Europe clearly addresses the need for action and emphasizes the role of national government agencies in overseeing the adoption and application of digital health, ensuring funding availability, and promoting health literacy and digital inclusion (19). Germany has also embarked on this path by developing national initiatives aimed at integrating digital tools into the healthcare domain (20). The objective of the present study was to explore the specific perceptions related to DHI among adolescents with T1DM residing in Germany. This study aimed to enhance the comprehension of DHI accessibility, their utilization, their roles, as well as their associations with health literacy. The results of this qualitative analysis are anticipated to provide further insights into DHI research, facilitating the promotion of health literacy and allowing for a targeted approach to address the specific requirements and actions required to align with national and international strategies in this domain.

## 2 Methodology

### 2.1 Research design

A qualitative approach was chosen to account for the exploratory nature of the study. Semi-structured individual interviews involving adolescents aged 14 to 18 years old with type 1 diabetes mellitus (T1DM) were considered as an appropriate method, especially for recruiting a hard-to-reach target group discussing potentially sensitive topics. Additionally, this method allowed for an in-depth understanding of participants’ experiences, including their perceptions and interpretations (21). The content analysis approach by Kuckartz et al. (22) was adopted to explore their perspectives on digital health interventions aimed at promoting health literacy. The study followed the Consolidated Criteria for Reporting Qualitative Research (COREQ) checklist developed by Tong et al. (23) to transparently report the study. The completed COREQ checklist, consisting of 32 items, is available and can be requested directly from the corresponding author of this study (AN.N).

### 2.2 Inclusion criteria and recruitment

The target population consisted of adolescents aged 14 to 18 years old with T1DM. An additional inclusion criterion was a command of the German language sufficient to allow for full participation in interviews. Moreover, participants under the age of 18 required informed consent from their parents in addition to their own informed consent. The recruitment process occurred in two phases. In the first phase, various recruitment methods were employed, such as distributing flyers in hospitals and specialized diabetes centers in different German cities, sending flyers via email to diabetes centers, disseminating information in support groups dedicated to young people with T1DM, as well as via online forums, official Instagram accounts of the T1DM community, Facebook groups, private requests on social media to influencers with T1DM and finally, through word-of-mouth. In total, 87 networks and institutions were approached. The second phase of recruitment took place during a six-week observation period at a pediatric diabetes center. The number of participants was two in the first phase and 18 in the second phase.

### 2.3 Data collection and processing

Semi-structured individual interviews were conducted. The first interview was conducted by phone, while the second took place via video conference. Subsequently, a total of 18 interviews were conducted within the pediatric diabetes department of a hospital in Germany. The first two interviews occurred between November and December 2022, and the remaining 18 were conducted between May and June 2023. All interviews were conducted by a single researcher, AN.N, a doctoral candidate experienced in qualitative interviews. It should be noted that one of the interviewees had a familial connection to an acquaintance of the researcher; the other 19 participants were unknown individuals prior to data collection. Before starting the interview with the recording, the researcher created a relaxed atmosphere, considered crucial as a “warm-up” by Reinders (24), to make the participants feel comfortable during the interview. Once situated in the room where the interview was to take place, the researcher reiterated the study’s purpose and the interviewee’s rights during and after the interviews. The introduction phase also allowed participants to ask questions. The interview was conducted using a semi-structured interview guide with open-ended questions to ensure that all themes were addressed while maintaining flexibility and adaptability for each interview. The interview guide was created following Reinders’ (24) structure and was divided into seven parts:

1. The “Warm-Up-Phase,” individualized for each interview, following Reinders (24)
2. Peer relationship
3. Communication between physicians and adolescents with T1DM
4. Training
5. Access to information more generally
6. Open-ended questions
7. Conclusion, socio-demographic data

The interview guide is available in the Supplementary material.

The interviews were recorded as digital audio recordings and transcribed in full by AN.N. Furthermore, field notes were taken after each interview, including observations and impressions. Age and the number of years since adolescents were diagnosed with T1DM were requested during the interview. Education, nationality, and gender were not requested. Gender was classified based on the researcher’s perception.

### 2.4 Data analysis

The interview guide was initially deductively coded inspired by the definitions of health literacy of Bröder et al. (25) and Naef et al. (18). Before commencing the study, a pilot test was conducted with two adolescents with T1DM. For this current study, a total of 20 adolescents (8 girls and 12 boys) with T1DM living in Germany were interviewed. Participants’ ages ranged from 14 to 18 years with an average age of 16 years. The authors did not provide the interview guide containing the questions to the interviewees. Interviews lasted between 17 and 33 min, with an average duration of 23 min. The adolescents had been living with T1DM for an average of 7 years, with a range of 6 months and 16 years. Individual interviews were conducted in person (by AN.N), by phone, via video conference, and in face-to-face settings. The authors discussed and determined that saturation of sampling (26) had been achieved. The coding system was conducted iteratively using MAXQDA software (2022; VERBI Software GmbH, Berlin, Germany). The analysis was subsequently refined through an iterative process involving structural content analysis (22). Two authors (AN.N and N.F) consolidated 100% of the entire coding material to ensure data quality, consistency, validity, and reliability of results (22).

### 2.5 Ethics statement

The study design was approved by the ethics committee of the Hannover Medical School on October 18, 2022. Information about the study was communicated orally and in writing to adolescents, and their parents in the case of minors, before the interview. Adolescents were informed that their participation was entirely voluntary, independent of the institutions where they received treatment, and the researcher emphasized multiple times during the interview that adolescents had the right not to answer questions or to terminate the interview without providing a reason. Adolescents were also informed that they could contact the researcher in the week following the interview if they wished for their interview not to be considered for the study. Interviews were audio-recorded and transcribed, after which the recordings were deleted. To ensure anonymity, any information that could identify participants was removed from the transcripts. All participants provided written consent for their participation and digital voice recording with additional written consent from parents or legal guardians obtained for minors. Participants did not receive vouchers or money for participating in the study. Personal data was processed in accordance with the General Data Protection Regulation and the Declaration of Helsinki.

## 3 Results

The coding system comprises 11 main categories and 26 subcategories (see Supplementary material). To address the research question, three main themes were analyzed in detail. The first theme focuses on access to information between digital and non-digital sources. The second theme examines digital interventions for peer relationships. Finally, the third theme explores the use of digital interventions for communication and interaction during medical consultations, outside of consultations, and in a hospital setting (see Table 1: Summary of the main results). The cited sources have been translated from German to English (AN.N & NF).

**Table 1:** Summary of the main results.

Information	Source of information: training programs, rehabilitation programs, consultations (non-digital) Parents as a source of information (non-digital) Manufacturers as a source of information (digital)
Peer-to-peer	Online forums and social media do not play a major role in peer relations ➔ lack of information about existing platforms and little interest Parents for parents Friends, family or other contact person / confidants play an important role, not only to get information, but for emotional support
Communication and interaction between patient and physician	Little use of digital interventions Different perspective about the use of digital interventions E-mails not used by adolescents ➔ perceived as a not efficient and non-secure Communication and interaction happen between parents and physicians, excluding patients

### 3.1 Access to information

Three primary sources of information regarding disease management were highlighted by the interviewees. The first encompasses all official information sources directly from institutions where adolescents with T1DM are being cared for. This includes those receiving inpatient care with an intensive training program, those participating in occasional training programs conducted in group or individual settings, as well as those attending REHA facilities (rehabilitation institutions) either on an inpatient or outpatient basis. Additionally, it encompasses information obtained during outpatient appointments during regular consultations with experts, including physicians and/or diabetologists. Information from these sources is primarily conveyed in person, with digital tools used only in rare cases. The second primary source of information highlighted was parents or close family members. While T1DM primarily manifests in minors, or family member may also be affected by the disease as T1DM is a genetic condition. The third primary source of information identified was the manufacturers of digital tools, such as pumps or sensors. Manufacturers play a significant informative role, mainly through digital channels like online chat features.

Initially, the primary sources of information for adolescents with T1DM in Germany are accessible during training sessions/programs that take place during the initial diagnosis. Adolescents spend an average of 2 weeks in inpatient care and receive information about disease management during an intensive training, often with the involvement of parents. These training sessions are conducted on-site and organized by the hospital. Specific, tailored training can occur either individually or in groups, with the majority being conducted in person, with a few exceptions, such as during the COVID-19 pandemic. Furthermore, the German healthcare system allows patients to undergo inpatient or outpatient rehabilitation (REHA) for several weeks, accompanied by one or both parents. Finally, regular outpatient hospital appointments, typically four times a year, also serve as a source of information for adolescents with T1DM, and are mainly conducted in person. As regards this type of official information dissemination (via hospital or institutions), some adolescents mentioned being overloaded with information:

"*It was too much. And getting everything explained in general, because it was all, everything intertwined, so many topics were covered simultaneously here.*" (P15:77)

This mass of information received all at once led at least one interviewee to seek information on the internet. Another interviewee received a book to read the information herself, which had already been mentioned during the training sessions. This participant admitted that she had not read the book, mainly because there was too much information.

"*Okay, I'll be honest, I didn't really look at the book, it was way too much. I only did the exercise for calculation, just so I have some basic knowledge. But I didn't go through the book again.*" (P16:24)

This information overload also leads to forgetting some information. For some, printed materials serve as a useful resource to check information.

"*I had already learned it. It was somewhere in my head. So, partly, when it was explained to me again, it did come back to me, but I'm a very, very forgetful person. That's why I'm a bit afraid that if, for example, if I want to change something again, I'll have to ask again or get another booklet that tells me what I can change. I think I'd look into that again.*" (P16:106)

For some individuals, language difficulty, for example limited German language skills and/or lack of knowledge of specific medical jargon, adds a barrier to understanding the information. Others may not always feel comfortable asking questions, depending on the context. For example, in a situation where eight medical students were present during the consultation, it made the patient nervous and prevented her from asking a question because she did not understand what the doctor was saying.

"*Yes, sometimes I do dare to ask questions if it's something very complicated. But, for example, before, I was a bit unsure. I didn't dare to ask.*" (P16:102)

To seek more information or better understand information provided by healthcare experts, some adolescents use digital interventions such as YouTube or social media platforms like TikTok or Instagram. These sources are not directly recommended by healthcare experts. Videos are considered a more easily understandable digital tool by adolescents than text or online searches, where information filtering is considered complicated. Some adolescents do not verify the information found on the internet or social media (*“I do not discuss that with my doctor.”* (P17:48)), while others have mixed opinions about the quality of the information but still take it into account:

"*So, I wouldn't consider it a reliable source, but (…) So, if, for example, if I had very high values (.) Yeah, I don't know, if something strange were to happen and there was information on YouTube like, 'You should eat nuts or something.' I don't know. Then I would try that.*" (P18:51)

For others, in-person consultations provide an opportunity to ask questions and obtain information from healthcare experts. In some cases, young patients may come across information on the internet, like Instagram posts about someone using a closed-loop system, and take the opportunity to discuss it with their physicians during their next appointment.

"*Yeah, so, I did (.) bring it up during the next appointment, that I would like to try it, and so on. (.) And that's how I tried the Loop System last month.*" (P19:28)

Official training sessions organized by healthcare institutions are generally considered to be an overload of information, too complex, or difficult to understand due to the language used. Interviewees mention seeking information outside of official sources, primarily on the internet and social media platforms like YouTube, Instagram, or TikTok. Sometimes, this information is not subject to expert scrutiny; in other cases, adolescents may express criticisms regarding this information but still use it, and in yet other instances, the information is discussed during subsequent consultations with healthcare experts.

The second major source of information often used by interviewees is the knowledge of parents, who play a significant role in accessing information. This is mainly because T1DM largely affects children and adolescents, and most of the interviewed adolescents were diagnosed in childhood. In such cases, parents have played a major role in managing the disease and have become experts through training and experience. The situation remains similar for adolescents who were diagnosed later because young people are still minors, and parents continue to be heavily involved in training.

"*If I have a question, I'll quickly discuss it with my mother because she knows a lot, of course. I got this when I was five, so I probably didn't understand it all that well. And so, she explained it to me bit by bit, as I got older, a little more each time […] And yes, we still do quite a bit together.*" (P9: 52)

This is not always the case, especially for patients whose parents do not speak neither German nor English. In such cases, other family members can play an important role, as is the case with a brother in this example:

"*My brother speaks perfect German. My parents only know a few words, and he can understand better.*" (P10:130)

Furthermore, the third significant source of information is the manufacturers of technologies such as sensors and pumps, which many adolescents use. This source of information is often compared to efficient customer service.

"*They answer immediately. So, there is, how should I say it, like a customer service. So, let's say, first-level customer service, okay? There is also higher customer service that can answer many difficult questions. If the first-level has problems, the first one, then they forward it to better customer service.*" (P5:98)

Some adolescents clearly differentiate where to seek information between technical problems, directly from the manufacturers, and clinical questions, at the hospital. Others are more hesitant about navigating between information sources, for example, when asked if they would talk to a doctor if a technical problem occurred:

"*I don't think so. Because the app is from [Company] and not from the [Institution]. So, I believe you can only contact the company.*" (P7:51)

In this theme, “Access to Information,” the results shows that adolescents with T1DM often find the initial dissemination of information from official sources overwhelming, leading them to seek additional information online or through alternative means. Language barriers, hesitation to ask questions, and the influence of social media also shape their information-seeking behaviors. These adolescents frequently turn to their parents, who play a crucial role in managing T1DM, and also rely on manufacturers’ customer support for technical assistance.

### 3.2 Peer-to-peer relations

The relationship between peers (adolescents with T1DM) is relatively limited, whether through digital interventions or in-person interactions. One reason for this is that most adolescents are unaware of the opportunities for exchanging or communicating with peers, whether through digital interventions or otherwise. Some study participants argue that such contact is unnecessary and that they lack interest in engaging with peers, emphasizing that everyone’s experience with the disease is unique:

"*I'm mainly interested in my own affairs, like how my blood sugar is regulated, and ultimately, everyone has a different perception of the disease, and everyone sees it a bit differently […] So, I'm more focused on my diabetes.*" (P9:16)

In rare cases, a classmate or schoolmate may also have T1DM. However, these interactions are often brief and superficial, focusing on topics like whether they use specific technologies such as insulin pumps, the manufacturer’s brand, or their HbA1c values:

"*We talked about our measuring and pumping systems. She doesn't like the sensor system. I'm not a hundred percent sure, but I think she had a pump with a tube. […] And that's basically all we said about diabetes.*" (P9:10)

Some adolescents express an unmet desire to exchange experiences with peers:

"*But (…) Now (…) looking back (…) […] I would like to talk to others about how they experienced it.*" (P1:48)

For some, the idea of exchanging experiences with peers becomes more appealing when combined with another activity. For example, one interviewee expressed interest in a sailing camp for adolescents with diabetes, which they can join from the age of 16. The flyer for the camp was seen in the hospital corridor. The combination of learning to sail and interacting with peers seemed to attract their attention:

"*At first (…) to learn how to sail. But it would also be cool to spend time with people who also have diabetes, to see how they do it. And, you know, how they manage it in school, for example.*" (P18: 32)

Some prefer to talk to their close friends rather than unfamiliar peers. These close friends are knowledgeable about the disease and are considered a significant source of support:

"*My classmates also knew about it. And my closest friends, the ones I'm always with, I explained to them what they should look out for or symptoms, for example, if I get pale, they should ask if I want to check my blood sugar. I told them some things about symptoms of both hypoglycemia and hyperglycemia, and they really help me.*" (P16:131)

A reference person who can provide support in more challenging times can also be someone who does not have the disease. In this case, they are seen as offering emotional support:

"*I already know everything, and I don't necessarily need other people there. But simply having that emotional support could mean that people can throw in ideas or something like that. And just the fact that even if neither I nor the others have diabetes and stuff, you still don't feel so alone at the moment. […] But maybe that came up later in the conversation. Always having someone there who listens to me. Maybe also brings in their own thoughts so that it happens. Maybe we can discuss this here again. So, somehow, a reference person.*" (P19:88)

For others, communicating with peers of the same age who have the same disease is of value, allowing them to ask questions they might not ask an adult, such as their parents, perhaps due to tension within the family. They feel better understood by peers of their own age, without indicating a preference for in-person or digital contact:

"*I think it's important because they can understand. And you feel like you can relate to everything, and you get suggestions or something. So, if you ask someone who doesn't have diabetes, they can't make suggestions.*" (P2:90)

For others, the age of their peers does not seem to play a crucial role, either in information exchange or in more personal or emotional exchanges. However, the fact that others have the same disease, regardless of age, plays a significant role. These individuals with T1DM can be, for example, an older distant cousin, a teacher, an aunt, or an individual from a support group. In one instance, the interviewee received individual support from an adult at school:

"*I had assistance from the first grade to the sixth grade, and she was only with me at school, helping me. I built a very strong relationship with her, and it really (…) She was like an aunt to me, who just stood by me and understood me […] She also helped me deal with giving insulin injections outside. I always had a problem with that (.) Okay, so why do I want to pull this pump out now? Everyone will look at me strangely. I was always afraid of that. But she helped me normalize it.*" (P7:91-97)

Finally, individuals followed on social media without personal contact are considered peers and examples, whether for learning or community-building purposes, the latter of which reducing feelings of loneliness:

"*It's still different to see people who have it themselves, how they deal with it, rather than just being told by a doctor, who may know a lot about it but doesn't have it themselves. It's still different to have those experiential values. To have it and because, usually, the people are a bit older and have more experience, so that you can integrate it well or just not feel so alone.*" (P19:34)

In general, adolescents report that peer groups are more active for parents who exchange information with other parents, whether online or in face-to-face meetings, sharing insights into new technologies and more.

### 3.3 Communication and interaction between individuals and physicians

Digital tools are relatively underutilized in Germany, both for video conferences during regular outpatient consultations and for other tools related to scheduling appointments or asking questions outside of appointments. Some young individuals prefer in-person consultations, believing that personal contact is important, especially for receiving advice, and find it more pleasant:

"*So. That was different. Definitely. So, personal conversation is better. And you ask more questions, maybe get some better advice, and it's just more pleasant. But it worked over the phone.*" (P4:60)

Others would prefer online consultations because they live far from hospitals and believe it does not affect the quality of communication:

"*That would be better. I wouldn't have to spend 1.5 hours coming here. I could save a lot of time.*" (P6:134)

Some would opt for a combination of both:

"*Well, definitely come here once every six months. Just to discuss, check the values, and make sure everything is okay. But maybe, for example, suggest that we meet the other time and alternate between in-person and phone consultations. That way, people who are a bit busier or always there don't lose so much time, but can do it from home and save some stress or something.*" (P4:64)

Others, taking a pragmatic view of the situation, propose a combination of face-to-face and online contact points. This is because some tests or checks must be done in person, and they are also aware of the healthcare system’s billing requirements for health insurance:

"*Actually, quite good, especially when you drive for an hour that day. And if it's just about talking about the values, where I have them and she also has them in front of her, then it's actually unnecessary to drive extra. It's necessary for measurements. So, giving blood values, body weight […] So, I have to come once a quarter. So, I'm not exactly sure why, but I think it's because of the health insurance billing, that's necessary.*" (P17:90-92)

Outside of appointments, communication primarily occurs between parents and physicians or other experts via phone or email, regardless of the adolescent’s age. Most adolescents do not use these two means of communication, and some even consider them unreliable:

"*I find email a bit unreliable.*" (P4:82),

"*Well, I think it would work. But the experience I've had is that you overlook it very quickly. So, that you misunderstand it. Either a problem arises where it doesn't get through, and stuff like that.*" (P16:96)

Some adolescents would prefer other digital tools to facilitate communication, especially in time-sensitive situations where there is a risk of not getting answers to their questions:

"*I don't know of an example, but for instance, we had a situation where my values were really high one evening. All through the night, too. And then we called the next day. And if that could be solved more quickly with some kind of app, I don't know, I'm not familiar with it, but if it could be resolved more quickly, so you don't have to call, and then the doctor has something to do, but instead, I don't know, write something where you can see it right away or something like this. It would help me.*" (P4:76)

Some have drawn parallels with applications implemented by schools during the COVID-19 pandemic for course management. Consequently, they expressed a desire to have the option to use a similar type of application in the medical field. For instance, they wished to engage in direct chats with physicians for inquiries, rescheduling appointments, interacting with other individuals with T1DM, or swiftly perform calculations to determine whether certain foods are suitable for consumption or not, all in real-time.

In this section, the results show that adolescents, regardless of their age, are relatively less engaged in communication with physicians or experts outside of appointments. Preferences for in-person or digital consultations are highly heterogeneous, and technological ideas have been clearly expressed by the interviewees.

## 4 Discussion

This study and its results identify an urgent need for development within the space of digital intervention aimed at improving health literacy for adolescents with type 1 diabetes. Such digital interventions as those widely used by the target group have the potential to support adolescents as they navigate the management of this chronic disease during a vulnerable period of life. Based on literature reviews (18, 25), this study and its results allow a deeper understanding of the specific needs of those directly affected, i.e., adolescents with type 1 diabetes. This is a valuable perspective for healthcare systems to take into account.

The analysis and findings of this study have identified three dominant themes. The first theme pertains to information access and adolescent perspectives’ revealing diverse opinions on various sources of information, both digital and non-digital. The second theme, focusing on peer relationships, exhibits heterogeneous statements regarding the significance of peer relationships, but relatively uniform opinions concerning the lack of knowledge about existing digital structures and platforms. The third theme, concerning perceptions, opinions and wishes regarding communication and interaction between adolescents and physicians, highlighted the desire for new digital forms of communication.

Based on these results, the authors emphasize three key points for future reflection, research, and policy formulation. Firstly, it is essential to provide adolescents with T1DM better access to available digital health interventions from institutions and experts (such as physicians) to enhance their disease management alongside the information received in institutional settings. This accessibility should be paralleled by the promotion of digital health literacy to ensure adolescents are well-equipped to use digital tools optimally. Secondly, it is crucial for this digital offering to be tailored and personalized to individual patients, catering to their unique needs. Finally, strategies for integrating digital tools should be developed with the active participation of adolescents with T1DM to better address the needs of this population.

This study reveals that across the three analyzed themes (information access, peer exchange, and communication), that there is a lack of communication from experts and institutions regarding the availability and accessibility of digital interventions. Officially, access to information occurs either within institutions (such as in hospital settings during regular outpatient consultations or in inpatient or outpatient rehabilitation facilities), or through intensive training programs following guidelines such as the ISPAD, for example (27). The positive effects of these in-person official information sessions on patient engagement and motivation have been demonstrated (28, 29). However, despite the positive impact of this information, the study results show that an excess of information, overly complicated information, or the fact that adolescents hesitate to ask questions when they do not understand the information for various reasons can lead some adolescents to seek information through digital interventions (such as YouTube or other social media platforms or the internet); information that is not recommended by physicians and whose quality is not verified. To address this issue, action should be taken on two fronts: First, regarding information overload and poor comprehension, it is crucial to ensure patient understanding, particularly to enhance their health literacy. This could be achieved through techniques such as “Plain Language,” which avoids complicated language and medical jargon, “Teach-Back” to confirm understanding of provided information, “Shared Decision Making” to collaborate with patients in reaching a common decision, or “Chunk and Check,” a strategy that breaks down large amounts of information into smaller sections, facilitating comprehension and retention of essential information (30, 31). Secondly, as the study shows that due to poor understanding, adolescents seek information elsewhere without expert verification, access to digital interventions should be guided. It is important to emphasize that the effectiveness of digital health interventions for patients with T1DM, whether for clinical improvements (13, 32) or their impact on health literacy (18), is supported by scientific literature. In light of this situation, it is necessary to ensure that the use of digital tools for information seeking occurs in conjunction with institutional training. Additionally, digital offerings should be endorsed and recommended by experts and institutions to ensure the quality of available information. In addition to access to this offering, it is important to support the use of digital tools through the promotion of digital health literacy. Improving the accessibility and visibility of digital offerings and their guidance by experts applies not only to information access and comprehension but also to peer interactions and communication between physicians and patients. The peer relationship and the communication between the physicians and the patient are also seen as an important source of information.

Moreover, recommendations made by experts and institutions to patients for specific types of digital health interventions must be personalized and individualized, considering the diverse needs of adolescents with T1DM. Indeed, the study results demonstrate heterogeneous opinions regarding the needs and desires for digital tool use and their perspectives on usefulness, whether for information access and comprehension, peer interaction, or communication with physicians. The literature also emphasizes the need to be more patient-oriented and provide personalized approaches to better target patient needs (33–36).

Finally, the study highlights the need to develop tools that allow for the active participation of those concerned, in this case T1DM patients, in institutional strategic planning. It is crucial to involve patients in decisions made at the organizational level (37, 38).

The three points mentioned above, which can serve as the basis for future deliberations, research, and policy development, align with the guidelines outlined in the recent report from the World Health Organization Regional Office for Europe, which explicitly underscores the need for action and highlights the role of national government agencies in monitoring the adoption and implementation of digital health solutions, making funding available, and promoting digital health and literacy (World Health Organization, Regional Office for Europe) (19). Similarly, in Germany, as mentioned by the Federal Ministry of Health (20), digitalization offers immense potential but is currently underutilized. It not only needs to be leveraged more effectively but also in conjunction with the promotion of digital health literacy (39, 40). Improved utilization of digital health interventions must also comply with the General Data Protection Regulation (GDPR) (41), which often restricts possibilities.

One of the major strengths of this study is the inclusion of the hard-to-reach group represented by adolescents with T1DM. The majority of interviews (*n* = 18) in a hospital setting ensured a diversified sample and avoided snowball or convenience sampling effects, allowing for a wide range of perspectives and thus resulting in heterogeneous groups. The interviews provided rich information on the need and manner of integrating digital interventions to promote health literacy among adolescents with T1DM. Data saturation was achieved and approved by the co-authors of this study. The conducted interviews no longer revealed significant new information compared to previous ones, indicating content saturation (26). However, several limitations must be acknowledged. Firstly, the second part of recruitment (*n* = 18) was limited to one hospital, in Germany, to ensure in-person participation. Additionally, self-reflection on one’s own position is crucial in qualitative research, especially as an adult who may be perceived as an authoritative figure by adolescents. The author’s subjectivity is not denied and is utilized as productive resource in the research (42).

## Data availability statement

The raw data supporting the conclusions of this article will be made available by the authors, without undue reservation.

## Ethics statement

The studies involving humans were approved by The study design and the interview guide have been summitted to the ethics committee of Medical School Hannover and have been accepted the 18th of October 2022. Approval number: 10559_BO_K_2022. The studies were conducted in accordance with the local legislation and institutional requirements. Written informed consent for participation in this study was provided by the participants’ legal guardians/next of kin. Written informed consent was obtained from the minor(s)’ legal guardian/next of kin for the publication of any potentially identifiable images or data included in this article.

## Author contributions

AN: Conceptualization, Data curation, Formal analysis, Investigation, Methodology, Project administration, Resources, Software, Validation, Visualization, Writing – original draft, Writing – review & editing. NF: Conceptualization, Data curation, Formal analysis, Investigation, Supervision, Validation, Writing – review & editing. HT-G: Methodology, Supervision, Validation, Writing – review & editing. VA: Conceptualization, Supervision, Validation, Writing – review & editing.
