# Supplementary material for: Adolescents with type 1 diabetes’ perspectives on digital health interventions to enhance health literacy: a qualitative study
Source: Front Public Health. 2024 Feb 13;12:1340196. doi: 10.3389/fpubh.2024.1340196 (PMC10896973; doi:10.3389/fpubh.2024.1340196)
Supplement: Supplementary file 1 [file Data_Sheet_1.PDF]

Supplementary Material: Category system (analyzed main and secondary categories)

Main category	Subcategory
(M1) Peer-to-Peer	
	(M1.S1) Model
	(M1.S2) Friends as Peers
	(M1.S3) Parents to Parents
	(M1.S4) School
	(M1.S5) Family as Peers
	(M1.S6) No Peers
	(M1.S7) Others
(M2) Communication and interaction	
	(M2.S1) Face-to-face
	(M2.S2) Digital communication tools - Messenger service
	(M2.S3) Phone call
	(M2.S4) Digital communication tools - Emails
	(M2.S5) Digital communication tools – new ideas
	(M2.S6) Others
(M3) Health information – Structured programs	
	(M3.S1) Health information through structured program: online
	(M3.S2) Health information through structured program: during disease manifestation – intensive care
	(M3.S3) Health information through structured program: individual training
	(M3.S4) Health information through structured program: groups training
	(M3.S5) Health information through structured program: rehabilitation programs
	(M3.S6) Health information through structured program: others
(M4) Health literacy (Sørensen et al., 2012*)	
	(M4.S1) Access
	(M4.S2) Understand
	(M4.S3) Appraise
	(M4.S4) Apply
(M5) Challenges	
(M6) Motivation	
(M7) Transition	
(M8) Disease Acceptance	
(M9) Others	
	(M9.S1) Sport, driving license, etc.
	(M9.S2) Support of the parents
	(M9.S3) Techniques (Sensor, pump)
(M10) Information non provided	
(M11) Warm up	
(F) Facts codes	
	(F1.S1) Age of adolescents with T1DM

	(F1.S2) Onset of T1DM
	(F1.S3) Symptoms

* Sørensen K, Van den Broucke S, Fullam J, Doyle G, Pelikan J, Slonska Z, Brand H, (HLS-EU) Consortium Health Literacy Project European. Health literacy and public health: a systematic review and integration of definitions and models. *BMC Public Health* (2012) 12:80. doi: 10.1186/1471-2458-12-80
