# Supplementary material for: Adolescents with type 1 diabetes’ perspectives on digital health interventions to enhance health literacy: a qualitative study
Source: Front Public Health. 2024 Feb 13;12:1340196. doi: 10.3389/fpubh.2024.1340196 (PMC10896973; doi:10.3389/fpubh.2024.1340196)
Supplement: Supplementary file 2 [file Data_Sheet_2.PDF]

Supplementary Material: Interview Guide

The interview guide has been translated from German.

1. The "Warm-Up-Phase" (individualized for each interview)

How did the consultation go? Feel free to tell me about it.
What do you like to do outside of school? Feel free to tell me about it.
What are your hobbies? Feel free to tell me about them.
You've been through something difficult; feel free to tell me more about it.

2. Peer relationships

Are you in contact with other young people your age who are also affected by type 1 diabetes?
How did you meet?
How do you communicate? What digital tools do you use?
If you are in contact, what do you talk about?
Can you tell me what you get out of talking to someone who is going through the same thing as you?
How would you rate the quality of information you receive from these people?
How do you apply the information/tips you get from people you know?
Can you give me an example?
(If not): Would you like to get in touch with them? If yes, why?

3. Communication between physicians and adolescents with T1DM

How often do you have appointments here? Do you always see the same physician?
Can you tell me how the appointment goes?
Who else do you talk to at your appointments/clinic visits other than your physician?
What kind of questions do you ask? Can you give me an example?
If you had questions about your diabetes that you couldn't ask, what would you ask? Can you remember such a situation?
Do you understand what the physicians tell you? If not, what do you do? Can you think of such a situation?
Do you feel that you can follow what the physicians say or the information you receive?
Do you keep in touch with your physicians between appointments? If so, how does this contact take place?
Do you meet in person or through other digital means?
If you could choose how to communicate with your physicians outside of appointments, how would you do it?

4. Training

Have you ever attended a training session? Can you tell me what a training session is like?
Do you have individual training sessions? How does that work?
What do you think are the advantages and disadvantages of group training versus individual training?
Do you feel that the training sessions give you all the information you need to manage your condition?
What additional information do you need?
What digital tools are used in the training sessions?
What is your best memory? Can you tell me what you learned and what you remembered?
What would you like to see in a training session when thinking about digital interventions (or tools)?
Could the training sessions be held online? What would that look like? What do you think about this?

5. Access to information more generally

We have discussed information from acquaintances, during consultations, and in structured training sessions. What other sources of information do you use to gather information?
What do you think is the best way to retain the information you need to manage your condition?

6. Open-ended questions

Could you please tell me: When were you diagnosed with type 1 diabetes?
When you think about our conversation, is there anything that we haven't discussed that is important to you in this context?

7. Conclusion, socio-demographic data

Age, Gender, Education
